# IN VIVO ANALYSIS OF REPORTER ALLELES REVEALS INCREASED VARIABILITY OF GENE EXPRESSION IN CELLS AND ANIMALS WITH AGE

**DOI:** 10.1093/geroni/igz038.380

**Published:** 2019-11-08

**Authors:** Nikolay Burnaevskiy, Bryan Sands, Soo Yun, Alexander Mendenhall

**Affiliations:** University of Washington, Seattle, Washington, United States

## Abstract

As a major risk factor for a multitude of chronic diseases aging is being increasingly recognized as a necessary therapeutic target for preventive medicine. Yet, despite tremendous progress in our understanding of the genetic determinants of longevity, proximal causes of aging remain incompletely understood. In part, this may be due to a plethora of factors, such as various types of stochastic macromolecular damage that affect individual cells and individual animals. Indeed, recent studies point to an increase of cell-to-cell variability in gene expression within old tissues, supporting the idea that stochastic events contribute to the aging process. Therefore, more single-cell focused studies are needed for a complete understanding of biological aging. Here, we utilized quantitative microscopy for analysis of gene expression in individual aging cells, in vivo in C. elegans. Using transcriptional reporters, fluorescently tagged proteins and a quantitative analytical framework adapted from yeast, we have found that young C. elegans exhibit very little stochastic or signaling noise in gene expression. However, using quantitative microscopy, we directly observed dysregulation of gene expression with age in vivo. Specifically, the stoichiometric ratios of proteins that are tightly regulated among the youthful populace start deviating in a cell autonomous fashion. Importantly, we find that an increase of gene expression variation is a relatively early event in the aging of C. elegans, readily observed before median lifespan. Hence, we suggest that incoherent cell-to-cell variation in gene expression arising with age can be an immediate causal factor for age-related loss of robust tissue function.

